# Local verification of ceftriaxone gradient strip MIC testing for *Neisseria gonorrhoeae* after CLSI 2026 breakpoint changes

**DOI:** 10.1128/spectrum.02569-26

**Published:** 2026-10-06

**Authors:** Li Li, Zijie Tang, Jiang Pu

**Affiliations:** 1 Nantong Center for Disease Control and Prevention 680464, Nantong, China; 2 Laboratory Medicine Center, Affiliated Hospital of Nantong University 74567, Nantong, Jiangsu, China; Montefiore Medical Center and Albert Einstein College of Medicine, Bronx, New York, USA

**Keywords:** *Neisseria gonorrhoeae*, ceftriaxone, antimicrobial susceptibility testing, CLSI, agar dilution, gradient strip MIC, disk diffusion

## Abstract

CLSI M100, 36th edition (2026), lowered the ceftriaxone susceptible MIC breakpoint for *Neisseria gonorrhoeae* from ≤0.25 mg/L to ≤0.12 mg/L, classified 0.25 mg/L as intermediate, and removed ceftriaxone disk diffusion interpretive criteria. We conducted a single-center, retrospective method-comparison study of 111 consecutive, nonduplicate, culture-confirmed isolates with paired ceftriaxone gradient strip MICs, agar dilution MICs, and archived 30-µg disk diffusion zone diameters. Gradient strip readings were prespecified to be rounded upward to the next nominal twofold dilution for comparison with agar dilution. Standardized essential agreement within ±1 dilution was 111/111 (100.0%); because upward rounding can increase apparent essential agreement, essential agreement based on the original continuous readings was also assessed and was 102/111 (91.9%). Using a binary interpretation based on the revised susceptible breakpoint (≤0.125 mg/L, displayed by CLSI as ≤0.12 mg/L, vs >0.125 mg/L), gradient strip and agar dilution classifications agreed for all 111 isolates (100.0%). Complete CLSI 2026 S/I/R categorical agreement was 105/111 (94.6%). The six discrepancies were all minor errors in which gradient strip testing classified an isolate as intermediate, whereas agar dilution classified it as resistant; no major or very major errors occurred. Gradient strip testing identified 12 of 18 isolates classified as resistant by agar dilution, corresponding to 66.7% sensitivity for the resistant category. Both methods classified 28 of 111 isolates (25.2%) as having MICs above the revised susceptible breakpoint; this proportion represents a classification frequency in a method-comparison data set, not a regional prevalence estimate.

**IMPORTANCE:**

Clinical laboratories need practical approaches when antimicrobial susceptibility breakpoints change. For *Neisseria gonorrhoeae*, the 2026 CLSI revision lowered the ceftriaxone susceptible MIC breakpoint and removed ceftriaxone disk diffusion interpretive criteria. Using paired isolate-level results, this study evaluated a product-specific ceftriaxone gradient strip workflow against agar dilution and examined archived disk diffusion results using the former 2025 CLSI susceptible-zone threshold of 35 mm. After prespecified upward rounding to the reference twofold scale, standardized essential agreement was 100.0% and complete CLSI 2026 S/I/R categorical agreement was 94.6%; all six categorical discrepancies involved gradient strip intermediate results and agar dilution resistant results. The former disk diffusion threshold showed limited specificity in this historical exploratory analysis. These data provide a single-center assessment to inform local verification of gradient strip MIC testing and transition toward MIC-based ceftriaxone surveillance.

## INTRODUCTION

Antimicrobial-resistant *Neisseria gonorrhoeae* remains a priority for clinical microbiology laboratories and public health programs (1, 2). Resistance to penicillin, tetracycline, and fluoroquinolones has narrowed treatment options, leaving ceftriaxone central to empirical therapy in many settings (2–4). Surveillance systems such as WHO EGASP, CDC GISP, and Euro-GASP, therefore, monitor ceftriaxone MIC distributions because shifts in MICs may be important before categorical resistance becomes common (1, 5, 6). Recent surveillance and molecular epidemiology studies from China also show why local ceftriaxone MIC results should be interpreted alongside broader resistance surveillance rather than as stand-alone prevalence or outcome data (7, 8).

Ceftriaxone surveillance is sensitive to small changes around the susceptible breakpoint. Agar dilution remains the reference method for gonococcal MIC testing, but it is labor-intensive and difficult to implement routinely in many clinical laboratories (9, 10). Gradient strip MIC testing on appropriate gonococcal media is more feasible and produces a quantitative MIC result, although recent studies show that results can vary with the strip product, tested MIC range, medium, incubation conditions, and reading time (11–13). Disk diffusion is inexpensive, but it has limited resolution when isolates cluster near a low MIC breakpoint (11, 14). CLSI M100, 35th edition (2025), retained ceftriaxone disk diffusion interpretive criteria for *N. gonorrhoeae*; CLSI M100, 36th edition (2026), lowered the ceftriaxone susceptible MIC breakpoint from ≤0.25 mg/L to ≤0.12 mg/L, classified 0.25 mg/L as intermediate, and removed ceftriaxone disk diffusion interpretive criteria (9, 15).

This breakpoint revision raises practical questions for laboratories with historical ceftriaxone disk diffusion data and limited capacity for routine agar dilution. We, therefore, sought to quantify the reclassification of isolates after application of the revised MIC breakpoint, assess agreement between gradient strip and agar dilution MIC results under the revised CLSI categories, and explore the performance of the former disk diffusion threshold as a historical screening signal. These objectives were addressed in an exploratory method-comparison study using paired gradient strip MICs, agar dilution MICs, and archived disk diffusion zone diameters.

## RESULTS

### Isolate characteristics

The analysis set comprised 111 consecutive eligible, nonduplicate *N. gonorrhoeae* isolate records with paired gradient strip MIC, agar dilution MIC, and archived disk diffusion results. Ninety-nine records were from male patients and 12 from female patients; median age was 30 years (interquartile range [IQR], 22–41 years). Specimens were urethral secretion/swab (*n* = 89), vaginal secretion/swab (*n* = 11), urine (*n* = 10), and pus (*n* = 1). No pharyngeal or rectal isolates were available because those specimen types were not submitted to the laboratory during the study period. Once a pure culture had been recovered and confirmed, all specimen-origin groups underwent the same standardized MIC procedures. The specimen distribution reflects routine clinical submission and isolate availability (Table 1).

**TABLE 1:** Isolate characteristics and ceftriaxone breakpoint reclassification^*a*^

Characteristic or analysis item	Result
Total nonduplicate eligible isolate records	111
Sex distribution	Male, 99/111 (89.2%); female, 12/111 (10.8%)
Age	Median, 30 years (IQR, 22–41)
Specimen source	Urethral secretion/swab, 89 (80.2%); vaginal secretion/swab, 11 (9.9%); urine, 10 (9.0%); pus, 1 (0.9%)
Gradient strip MIC range (rounded twofold scale)	0.008–1 mg/L
Gradient strip MIC50/MIC90 (rounded twofold scale)	0.064/0.5 mg/L
Agar dilution MIC range (twofold scale)	0.004–1 mg/L
Agar dilution MIC50/MIC90 (twofold scale)	0.064/0.5 mg/L
30 µg ceftriaxone disk zone	Range, 20–43 mm; median, 33 mm (IQR, 30–36)
Gradient strip MIC >0.25 mg/L using former breakpoint	12/111 (10.8%)
Gradient strip MIC >0.125 mg/L (displayed as >0.12 mg/L) using revised breakpoint	28/111 (25.2%)
Gradient strip records newly reclassified	16/111 (14.4%)
Agar dilution MIC >0.25 mg/L using former breakpoint	18/111 (16.2%)
Agar dilution MIC >0.125 mg/L (displayed as >0.12 mg/L) using revised breakpoint	28/111 (25.2%)
Agar dilution records newly reclassified	10/111 (9.0%)

^*a*^ The main analysis retained all 111 consecutive nonduplicate isolate records after same-patient duplicate isolates were removed.

### Ceftriaxone MIC distributions, breakpoint reclassification, and gradient strip agreement

**Fig 1:**
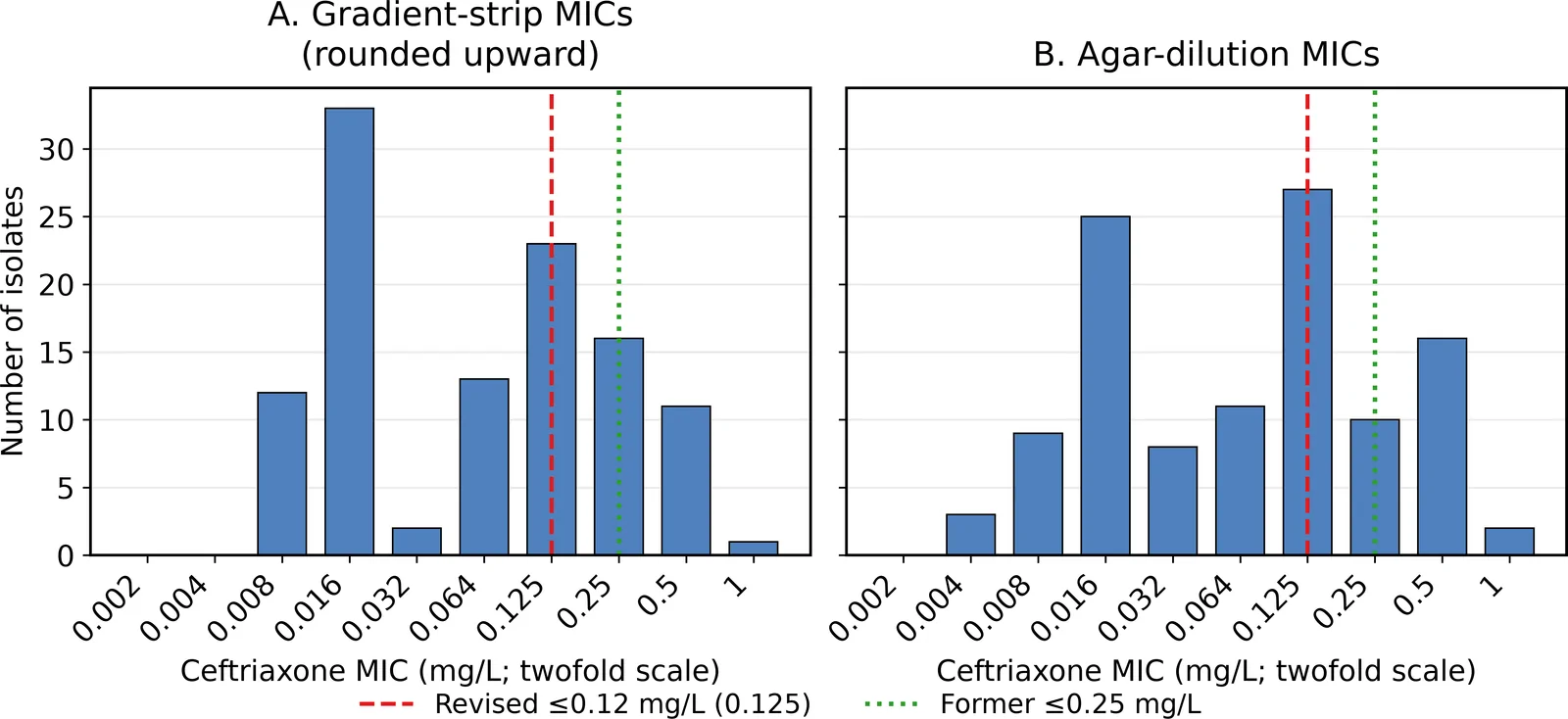
Ceftriaxone MIC distributions after the CLSI breakpoint revision. (**A**) Gradient strip MICs rounded upward to the next highest nominal twofold dilution in the agar dilution series. (**B**) Agar dilution MICs. Vertical lines indicate the former susceptible breakpoint of 0.25 mg/L and the revised experimental twofold threshold of 0.125 mg/L, displayed by CLSI as ≤0.12 mg/L. Bars show the number of isolates at each MIC concentration.

Following prespecified upward rounding to the twofold agar dilution series, MICs measured by gradient strips ranged from 0.008 to 1 mg/L, with MIC50 and MIC90 values of 0.064 and 0.5 mg/L, respectively. MICs determined by agar dilution ranged from 0.004 to 1 mg/L, with corresponding MIC50 and MIC90 values of 0.064 and 0.5 mg/L, respectively. The MIC distributions are shown in Fig. 1. The original continuous readings were retained for correlation analyses and the continuous-value analysis presented in Fig. S1.

The lower CLSI 2026 MIC breakpoint changed isolate classification. By gradient strip testing after upward rounding, 12/111 isolates (10.8%) had MICs above the former susceptible breakpoint of ≤0.25 mg/L, whereas 28/111 (25.2%) had MICs >0.125 mg/L; 16 isolates (14.4%) were newly reclassified. By agar dilution, 18/111 isolates (16.2%) had MICs above the former breakpoint, whereas 28/111 (25.2%) had MICs >0.125 mg/L; 10 isolates (9.0%) were newly reclassified (Table 1; Fig. 1). The 25.2% value is a classification frequency within this method-comparison data set.

**Fig 2:**
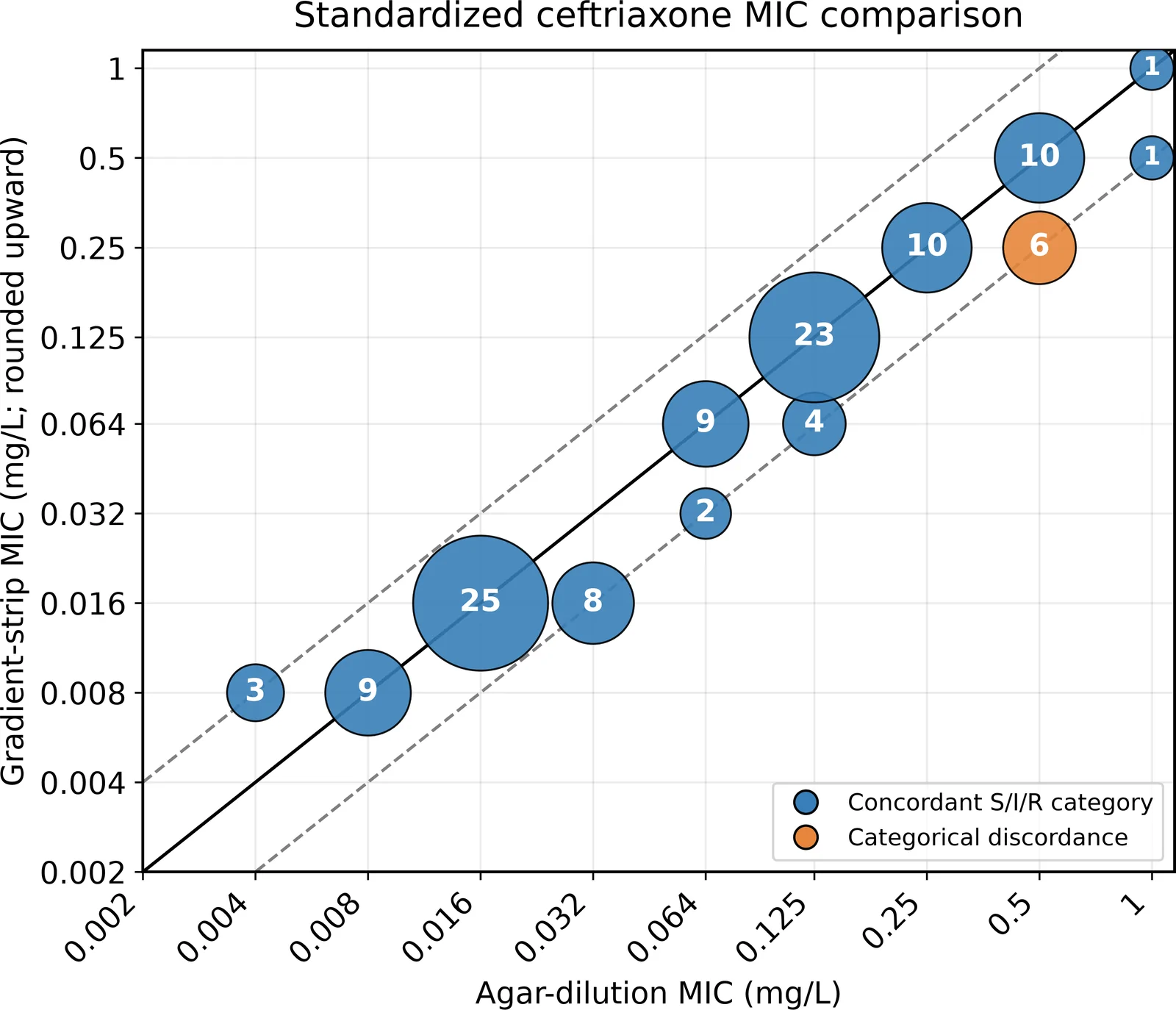
Standardized comparison of ceftriaxone gradient strip and agar dilution MICs. Gradient strip readings were rounded upward to the next highest nominal twofold dilution. Blue circles indicate concordant CLSI 2026 S/I/R categories, and orange circles indicate categorical discordance. The number printed inside each circle is the number of isolates at that MIC pair, and the circle size reflects overlapping records. The solid diagonal is identity; dashed lines indicate ±1 twofold dilution. Continuous raw readings and correlation are shown in Fig. S1.

After upward rounding, essential agreement within ±1 twofold dilution was 111/111 (100.0%; 95% confidence interval [CI], 96.7–100.0). Essential agreement based on the original continuous readings was 102/111 (91.9%; 95% CI, 85.3–95.7), indicating that upward rounding increased the apparent essential agreement. The nine original non-EA pairs all remained below the CLSI 2026 resistant breakpoint of ≥0.5 mg/L; both methods classified all nine isolates as susceptible, and none underwent categorical reclassification (Table 2; Tables S1 and S2). At the revised susceptible breakpoint, the two methods gave identical binary classifications for all 111 isolates (100.0%; 95% CI, 96.7–100.0). In the complete CLSI 2026 three-category comparison, gradient strip testing classified 83, 16, and 12 isolates as susceptible, intermediate, and resistant, respectively, whereas agar dilution classified 83, 10, and 18. Categorical agreement (CA) was 105/111 (94.6%). All six discrepancies were minor errors involving a gradient strip intermediate result and an agar dilution resistant result, indicating underestimation of the categorical level by gradient strip testing(Fig. 2). Among the 18 isolates classified as resistant by agar dilution, 12 were classified as resistant by gradient strip testing, corresponding to 66.7% sensitivity for the resistant category. No major errors (MEs) or very major errors (VMEs) were observed. Spearman correlation based on the original continuous readings was *ρ* = 0.975.

**TABLE 2:** Agreement between ceftriaxone gradient strip MIC and agar dilution MIC^*a*^

Gradient strip category (rounded)	Agar dilution category counts (S, I, R)
S	83/0/0
I	0/10/6
R	0/0/12
Complete three-category CA	105/111 (94.6%; 95% CI, 88.7–97.5)
Binary revised susceptible/elevated CA	111/111 (100.0%; 95% CI, 96.7–100.0)
Essential agreement (EA), ±1 dilution	111/111 (100.0%; 95% CI, 96.7–100.0)
Minor errors	6/111 (5.4%); all gradient I vs agar R
Major errors	0/83 (0.0%)
Very major errors	0/18 (0.0%)
Raw continuous pairs >1 dilution before rounding	9/111 (8.1%); all gradient readings lower
Spearman correlation, raw gradient vs agar MIC	*ρ* = 0.975

^*a*^ In the Table 2 matrix, each agar dilution count is ordered S, I, and R. EA was calculated after upward rounding of gradient strip readings to the agar dilution twofold series. CA, ME, VME, and minor errors refer to the complete CLSI 2026 S/I/R comparison; binary CA refers only to ≤0.125 mg/L vs >0.125 mg/L. The displayed CLSI breakpoint ≤0.12 mg/L corresponds to the experimental 0.125 mg/L dilution.

### Performance of the former disk diffusion threshold

Among the 111 archived results, 66 isolates (59.5%) had zone diameters <35 mm, below the former susceptible-zone cutoff of ≥35 mm. Using the revised agar-dilution MIC classification as the reference, the <35 mm screen identified 23 of 28 isolates with MICs >0.125 mg/L and 43 of 83 isolates with MICs ≤0.125 mg/L; 40 of the latter isolates and 5 of the elevated-MIC isolates were screen-negative (Table 3). These labels describe the historical rule only; CLSI M100, 36th edition, provides no current ceftriaxone disk-diffusion categories for *N. gonorrhoeae*. The historical <35 mm screen had 82.1% sensitivity (95% CI, 64.4–92.1) and 48.2% specificity (95% CI, 37.8–58.8) for detecting agar-dilution MICs >0.125 mg/L.

**TABLE 3:** Former ceftriaxone disk diffusion screen-positive (<35 mm) threshold vs agar dilution MIC classification^*a*^

Analysis item	Result
Agar MIC >0.125 mg/L (displayed as >0.12 mg/L; *n* = 28)	Zone < 35 mm: 23; zone ≥35 mm: 5
Agar MIC ≤0.125 mg/L (displayed as ≤0.12 mg/L; *n* = 83)	Zone <35 mm: 43; zone ≥35 mm: 40
Total	Zone <35 mm: 66; zone ≥35 mm: 45
Sensitivity	82.1% (95% CI, 64.4–92.1)
Specificity	48.2% (95% CI, 37.8–58.8)
Positive predictive value	34.8% (95% CI, 24.5–46.9)
Negative predictive value	88.9% (95% CI, 76.5–95.2)
False-positive rate	51.8% (95% CI, 41.2–62.2)
False-negative rate	17.9% (95% CI, 7.9–35.6)
Spearman correlation, zone vs agar MIC	*ρ* = −0.644

^*a*^ The former <35 mm rule was a historical binary screen and is not a current CLSI 2026 ceftriaxone disk diffusion interpretive method. Denominators are defined by the agar dilution reference classification.

The former susceptible-zone cutoff captured most agar-dilution isolates newly reclassified by the revised breakpoint but also flagged many MIC-susceptible isolates. Among the 10 agar-dilution isolates newly reclassified by the revised breakpoint, 9 (90.0%) had zone diameters <35 mm. By contrast, 43 of 83 isolates with agar-dilution MICs ≤0.125 mg/L had zone diameters <35 mm. Among the 18 isolates with agar-dilution MICs >0.25 mg/L, 14 (77.8%) had zone diameters <35 mm.

### Zone diameter-MIC relationship

**Fig 3:**
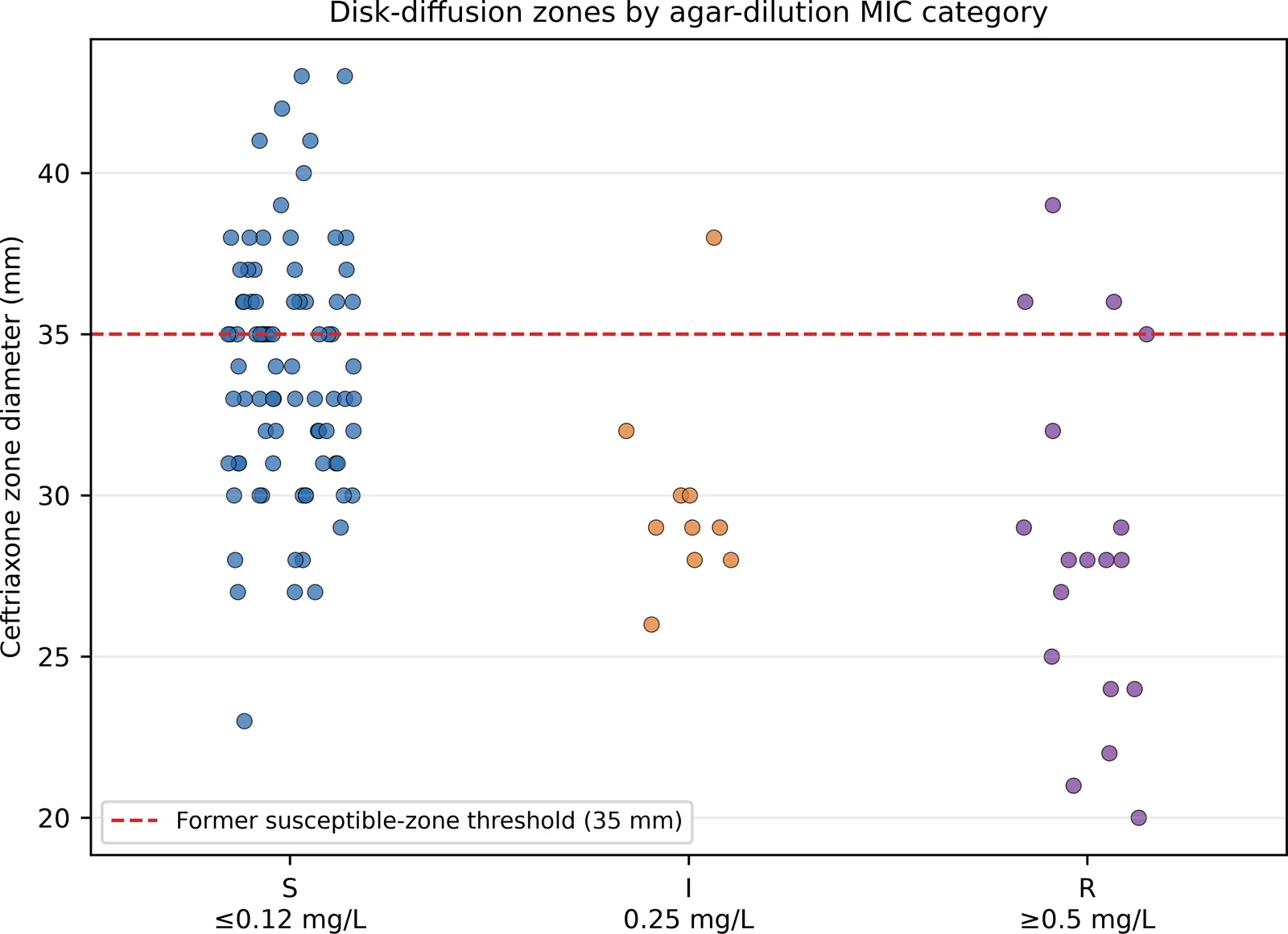
Ceftriaxone disk diffusion zone diameters by agar dilution MIC category. Points show individual isolates. The dashed horizontal line marks the former 2025 susceptible-zone threshold of 35 mm. The <35 mm rule is shown only as a historical exploratory screen because CLSI M100, 36th edition, removed ceftriaxone disk diffusion interpretive criteria for *Neisseria gonorrhoeae*.

Archived disk diffusion zone diameters were generally smaller among isolates with agar dilution MICs above the revised susceptible breakpoint than among those at or below the breakpoint, although substantial overlap was observed between groups (Fig. 3). Detailed zone diameter distributions and the Spearman correlation with agar dilution MICs are provided in the Supplemental Material.

## DISCUSSION

This study provides two laboratory messages after the CLSI 2026 ceftriaxone revision for *N. gonorrhoeae*. First, a lower susceptible MIC breakpoint changes surveillance classification without changing the measured MIC. Second, a product-specific gradient strip workflow can be locally verified against agar dilution, whereas the former disk diffusion threshold is an exploratory historical signal with limited specificity and no current CLSI categorical status.

Published China Gonococcal Resistance Surveillance Programme (China-GRSP) studies describe both laboratory coordination and quality assurance. In the 2013–2016 analysis, isolates were transferred to provincial central sexually transmitted disease laboratories for agar dilution testing according to WHO recommendations. All participating central laboratories took part in the WHO Western Pacific GASP external quality assurance program through the National STD Reference Laboratory in Nanjing (16). A subsequent study of China-GRSP isolates collected in 2018 used WHO *N. gonorrhoeae* reference strains G, J, K, L, and P together with ATCC 49226 for quality assurance and included testing batches when reference-strain MICs were within the reference ranges (17).

Relevant Chinese diagnostic and clinical guidance includes WS/T 268–2019 (Gonorrhea diagnosis) and the 2020 national guideline for the diagnosis and treatment of syphilis, gonorrhea, and genital *Chlamydia trachomatis* infection (18, 19). In China-GRSP surveillance, decreased susceptibility was defined as MIC ≥ 0.125 mg/L in the 2013–2016 analysis (16) and as MIC ≥ 0.25 mg/L in the study of isolates collected in 2018 (17). A subsequent national report of ceftriaxone-resistant gonorrhea further illustrates the need to interpret surveillance estimates in relation to the testing method and operational threshold used (20). These period-specific surveillance definitions should not be treated as a single immutable national clinical breakpoint and should be distinguished from the revised CLSI M100, 36th edition, clinical breakpoint.

The 2026 breakpoint revision has direct implications for clinical reporting and surveillance. WHO EGASP and Chinese gonococcal surveillance programs generally rely on culture-based susceptibility testing and quantitative MIC distributions; however, surveillance estimates should not be directly compared or pooled when specimen composition, isolate selection, testing method, or breakpoint standard differs. Chinese national surveillance reports should, therefore, specify the testing method and interpretive standard used for each reporting period. Our single-center method-comparison data set was not sampled according to a population-weighted national surveillance design. Apparent changes in the proportion of susceptible isolates after the 2026 revision may, therefore, reflect reclassification rather than a biological increase in resistance. The breakpoint revision does not by itself warrant an immediate change in empirical ceftriaxone therapy. Treatment should follow current Chinese and WHO clinical guidance, taking into account the infection site, local and national surveillance data, pharmacokinetic/pharmacodynamic evidence, and documented treatment outcomes.

After standardized rounding, gradient strip testing achieved 100.0% essential agreement and 94.6% complete S/I/R categorical agreement with agar dilution; binary revised susceptible/elevated classification agreed in all 111 isolates. The original continuous-reading essential agreement was 91.9%, and upward rounding can increase apparent agreement. All six categorical discrepancies involved gradient strip intermediate results and agar dilution resistant results, indicating a lower categorical classification by the gradient method. These findings support a pragmatic, product-specific MIC workflow in this laboratory after local agar dilution verification. Breakpoint-near and elevated-MIC isolates should be repeated or referred to a reference laboratory when the result is unexpected, discordant, or associated with suspected treatment failure.

A practical transition can be organized according to laboratory capacity rather than by requiring every site to establish agar dilution immediately. Laboratories without routine MIC capacity should continue culture and species identification, record the exact disk-zone diameter and testing metadata, and report the result only as a noncategorical screening observation. The former <35 mm rule should not be converted into a current S/I/R result. A low zone may prompt repeat culture, isolate preservation, and prioritization for referral according to local capacity, but it cannot alone establish a current ceftriaxone category. Persistent quality-control failure, an unexpected clinical result, or suspected treatment failure should trigger referral to the designated provincial or national reference laboratory.

Where gradient-strip testing is feasible, laboratories should verify the specific product locally against agar dilution using isolates spanning low, breakpoint-near, and elevated MICs. Verification should include repeat testing across relevant days or operators, predefined acceptance criteria based on CLSI M52 or ISO 20776–2, routine use of ATCC 49226, and documented review of results at 0.125 or 0.25 mg/L and at or above 0.5 mg/L (21, 22). Discordant results, MICs at or above 0.25 mg/L, or results associated with suspected treatment failure should be referred rather than resolved by applying the former disk rule. During implementation, reports should include the MIC, method, and interpretive standard. When no MIC is available, the exact zone diameter should be reported with an explicit statement that CLSI provides no current ceftriaxone disk-diffusion category for *N. gonorrhoeae*.

At the network level, provincial central laboratories can provide confirmatory agar dilution, distribute standard operating procedures, train local staff, review referred isolates, and coordinate external quality assurance with the National STD Reference Laboratory and WHO reference strains, consistent with reported China-GRSP practice (16, 17). Laboratories without full agar-dilution capacity can submit triggered isolates and periodic subsets for proficiency monitoring. This capacity-based model allows quantitative MIC surveillance to expand while linking local testing with centralized confirmation and quality assurance. However, implementation may be constrained by transport costs, delays in isolate referral and confirmatory testing, and limited staff capacity at participating laboratories. Its feasibility will depend on reliable isolate transport, timely feedback from reference laboratories, and sustained local participation; these operational factors were not evaluated in the present study.

The former 2025 disk-diffusion rule used a susceptible-zone cutoff of ≥35 mm, with zone diameters <35 mm classified as below the susceptible threshold. This rule was developed alongside the previous ceftriaxone susceptible MIC breakpoint of ≤0.25 mg/L and cannot be transferred directly to the revised susceptible interpretation of ≤0.12 mg/L or to the new S/I/R categories. Zone diameters overlapped substantially across isolates classified by the revised MIC categories. The former threshold should, therefore, be used only to describe historical results or assess retrospective trends, whereas current categorical reporting should be based on MIC results.

Archived disk results were not generated at the same time as gradient strip and agar dilution testing and did not use the same inoculum suspension. Differences in isolate growth state, inoculum preparation, media and disk batches, incubation conditions, operators, and endpoint reading may have affected zone diameters and the calculated performance estimates. Accordingly, the disk analysis should be interpreted as an exploratory assessment of a historical rule.

The sampling frame was limited: most isolates came from male patients and urethral specimens; only 12 came from female patients, and no pharyngeal or rectal isolates were available. Specimen-specific pre-culture recovery was outside the scope of this isolate-level comparison, so culture yield and national prevalence could not be estimated. Routine quality control used only *N. gonorrhoeae* ATCC 49226; broader WHO reference strain panels and performance across the full MIC range were not assessed. The single-center, single-product design and limited product traceability in the retrospective records further restrict generalizability. Clinical outcomes, including treatment failures, molecular determinants, inter-reader variability, and replicate MIC measurements for every isolate, were unavailable.

In conclusion, applying the CLSI 2026 ceftriaxone MIC breakpoint reclassified a substantial fraction of isolates in this method-comparison data set. After upward rounding to the reference dilution scale, the Autobio ceftriaxone gradient strip workflow showed 100.0% essential agreement and 94.6% complete S/I/R categorical agreement with agar dilution, with no major or very major errors; resistant-category sensitivity was 12/18 (66.7%). The original continuous-reading essential agreement was 91.9%, and the six categorical discrepancies all represented gradient strip intermediate results paired with agar dilution-resistant results. These results support local MIC workflow verification, not a universal validation claim. The former <35 mm disk diffusion threshold had limited specificity and should not be used as a current CLSI categorical result or as a new clinical breakpoint. Longitudinal gonococcal surveillance should retain quantitative MICs and annotate method and breakpoint changes.

## MATERIALS AND METHODS

### Study design and data source

This exploratory, retrospective method comparison included consecutive eligible, culture-confirmed *N. gonorrhoeae* isolates recovered at a tertiary teaching hospital from January 2025 to March 2026. Only the first isolate from each patient was eligible, provided an archived ceftriaxone disk diffusion result was available, and the isolate remained viable for recovery and MIC testing. Repeat patient isolates and those that could not be recovered or reliably confirmed by VITEK MS (bioMérieux) were excluded. Before MIC testing, recovered isolates were subcultured for fresh, pure growth and reconfirmed by VITEK MS. Isolates from urethral, vaginal, urine, and pus specimens underwent the same MIC workflow. Original clinical specimens were unavailable; specimen collection, transport, and specimen-specific pre-culture processing were outside the scope of this post-recovery comparison. Records were deidentified before analysis.

### MIC methods, index method, and quality control

The Autobio Diagnostics ceftriaxone gradient strip (Zhengzhou, China; NMPA registration no. 豫械注准20142400170) was the index method. Agar dilution was the reference method for MIC comparison and analysis of archived disk diffusion results.

For agar dilution, ceftriaxone sodium reference standard (Yuanye Bio-Technology, China) was incorporated into GC agar base with 1% defined growth supplement at twofold concentrations from 0.002 to 1 mg/L. Approximately 10⁴ CFU were inoculated onto each spot. Plates were incubated at 35°C ± 1°C in 5% ± 1% CO₂ and 70%–80% humidity for 20–24 h. The MIC was the lowest concentration that completely inhibited visible growth under suitable illumination (10).

Gradient strip testing used the same medium and laboratory-defined incubation conditions because the manufacturer’s instructions did not specify separate incubation conditions for *N. gonorrhoeae*. Following the manufacturer’s reading instructions for bactericidal agents, endpoints were read at complete inhibition of visible growth, including hazy growth, microcolonies, and single colonies; colonies within 1–3 mm of the strip were not disregarded. If the intersection fell between concentration marks, the higher value was recorded. When readings from the two sides differed, the higher value was used; a difference greater than one dilution prompted repeat testing.

No distinct colonies were observed within otherwise clear inhibition ellipses among the 111 isolates, and none required exclusion or adjudication on this basis. Had such colonies occurred, they would have been subcultured, reconfirmed by VITEK MS, and subjected to repeat gradient strip testing from a fresh pure culture.

*N. gonorrhoeae* ATCC 49226 was the routine quality-control strain for each agar dilution batch. All recorded results were within the applicable CLSI quality-control ranges.

### Historical ceftriaxone disk diffusion procedure (2025)

Archived disk diffusion results were generated according to CLSI M100, 35th edition (2025), for *N. gonorrhoeae* (14, 15). Testing used GC agar base with 1% defined growth supplement. A direct colony suspension from fresh growth was adjusted to 0.5 McFarland, inoculated onto the agar surface, and tested with a 30-µg ceftriaxone disk. Plates were incubated at 35°C ± 2°C in 5% CO₂ for 20–24 h. Zone diameters were measured to the nearest millimeter at complete inhibition. Disk testing was not contemporaneous with the paired MIC measurements.

### Local verification and result review

All 111 isolates had paired gradient strip and agar dilution MIC results. Local verification considered quality-control results and the prespecified agreement and error criteria. Breakpoint-near and discordant pairs were reviewed to confirm endpoint interpretation and data recording without altering either method’s original result.

### Interpretive criteria

The former CLSI ceftriaxone susceptible MIC breakpoint was ≤0.25 mg/L (M100, 35th edition, 2025). The revised susceptible breakpoint is displayed as ≤0.12 mg/L (M100, 36th edition, 2026), corresponding to the 0.125 mg/L twofold concentration in the laboratory series. Accordingly, isolates were categorized as susceptible (S; MIC ≤ 0.125 mg/L), intermediate (I; 0.25 mg/L), or resistant (R; ≥0.5 mg/L). Binary analyses distinguished MICs at or below the revised susceptible breakpoint from those above it; MICs >0.125 to ≤0.25 mg/L were newly reclassified by the revision.

For the historical disk comparison, zones <35 mm were below the former susceptible threshold, and zones ≥35 mm met it. These labels were used only for retrospective exploratory analysis and did not represent current susceptibility categories because CLSI M100, 36th edition, removed ceftriaxone disk diffusion interpretive criteria for *N. gonorrhoeae*.

### Outcomes and statistical analysis

The prespecified outcomes were breakpoint-related reclassification, agreement between gradient strip testing and agar dilution, and performance of the former disk threshold for detecting MICs above the revised susceptible breakpoint. Method-comparison metrics were selected with reference to CLSI M52 and ISO 20776–2 performance-evaluation principles (21, 22).

For standardized analysis, a prespecified, study-defined ceiling conversion assigned gradient strip readings between nominal twofold concentrations to the next higher concentration; values already on the scale were unchanged. For example, 0.012 mg/L became 0.016 mg/L. Rounded values were used for primary agreement metrics, categorical errors, S/I/R categorization, and MIC distributions. Original readings were retained in Table S1 and Fig. S1.

Essential agreement (EA) was a rounded gradient strip MIC within ±1 twofold dilution of agar dilution. EA was also calculated from original readings to assess the effect of rounding. Categorical agreement was assignment to the same complete CLSI 2026 S/I/R category. A ME was a gradient strip resistant result paired with an agar dilution susceptible result; a VME was the reverse. A minor error involved susceptible vs intermediate or intermediate vs resistant results. Binary agreement at the revised susceptible breakpoint was summarized separately.

MIC_50_ and MIC_90_ were calculated from the ordered MIC distributions. Categorical variables were summarized as counts and percentages, and continuous variables as medians with interquartile ranges or ranges. Disk diffusion sensitivity, specificity, positive and negative predictive values, and false-positive and false-negative rates were calculated using the revised binary agar dilution classification as the reference. Wilson 95% confidence intervals were calculated for proportions. Spearman correlations compared original gradient strip readings with agar dilution MICs and zone diameters with agar dilution MICs. No multivariable model was fitted because the data set was small and exploratory.

## Data Availability

The deidentified isolate-level data supporting this article are provided in Table S1.

## SUPPLEMENTAL MATERIAL

Supplemental figure legend spectrum.02569-26-s0001.tif Legends for Fig. S1.

Fig. S1 spectrum.02569-26-s0002.docx Continuous ceftriaxone gradient strip MIC readings versus agar dilution MICs. Each point represents one isolate using the original continuous gradient strip reading; the solid diagonal line indicates identity. This figure is provided as a continuous value complement to the standardized, rounded upward comparison in Fig. 2. Spearman correlation was ρ = 0.975.

Tables S1 to S3 spectrum.02569-26-s0003.docx Standardized MIC distribution, agreement, categorical, and error analyses use gradient strip MICs rounded upward to the agar dilution twofold series. Raw continuous readings are retained in separate columns for correlation and Supplemental Figure S1; standardized agreement and categorical analyses use the rounded upward twofold values.

ASM does not own the copyrights to Supplemental Material that may be linked to, or accessed through, an article. The authors have granted ASM a non-exclusive, world-wide license to publish the Supplemental Material files. Please contact the corresponding author directly for reuse.
