# Supplementary material for: Local verification of ceftriaxone gradient strip MIC testing for Neisseria gonorrhoeae after CLSI 2026 breakpoint changes
Source: Microbiol Spectr. 2026 Oct 6;14(11):e02569-26. doi: 10.1128/spectrum.02569-26 (PMC13729158; doi:10.1128/spectrum.02569-26)
Supplement: Supplementary file 1 [file spectrum.02569-26-s0002.docx]

Supplemental Figure Legends

Supplemental Figure S1. Continuous ceftriaxone gradient strip MIC readings versus agar dilution MICs. Each point represents one isolate using the original continuous gradient strip reading; the solid diagonal line indicates identity. This figure is provided as a continuous value complement to the standardized, rounded upward comparison in Fig. 2. Spearman correlation was ρ = 0.975.
