# Supplementary material for: Local verification of ceftriaxone gradient strip MIC testing for Neisseria gonorrhoeae after CLSI 2026 breakpoint changes
Source: Microbiol Spectr. 2026 Oct 6;14(11):e02569-26. doi: 10.1128/spectrum.02569-26 (PMC13729158; doi:10.1128/spectrum.02569-26)
Supplement: Supplementary file 2 [file spectrum.02569-26-s0003.docx]

Supplemental Material

Supplemental material for: Local verification of ceftriaxone gradient strip MIC testing for *Neisseria gonorrhoeae* after CLSI 2026 breakpoint changes

Standardized MIC distribution, agreement, categorical, and error analyses use gradient strip MICs rounded upward to the agar dilution twofold series. Raw continuous readings are retained in separate columns for correlation and Supplemental Figure S1; standardized agreement and categorical analyses use the rounded upward twofold values.

Table S1. Paired ceftriaxone MIC and disk diffusion results for 111 consecutive nonduplicate *N. gonorrhoeae* isolates.

| **Study ID** | **Sex** | **Age** | **Specimen** | **Gradient raw MIC (mg/L)** | **Gradient rounded MIC (mg/L)** | **Agar dilution MIC (mg/L)** | **Raw log2 difference** | **Within ±1 dilution after rounding** | **30-µg disk zone (mm)** | **Gradient S/I/R** | **Agar S/I/R** |
| --- | --- | --- | --- | --- | --- | --- | --- | --- | --- | --- | --- |
| NG001 | m | 19 | Urethral secretion/swab | 0.012 | 0.016 | 0.016 | -0.42 | Yes | 35 | S | S |
| NG002 | f | 26 | Vaginal secretion/swab | 0.016 | 0.016 | 0.016 | 0.00 | Yes | 34 | S | S |
| NG003 | m | 29 | Urethral secretion/swab | 0.012 | 0.016 | 0.032 | -1.42 | Yes | 34 | S | S |
| NG004 | m | 20 | Urethral secretion/swab | 0.064 | 0.064 | 0.064 | 0.00 | Yes | 32 | S | S |
| NG005 | m | 27 | Urethral secretion/swab | 0.094 | 0.125 | 0.125 | -0.41 | Yes | 31 | S | S |
| NG006 | m | 24 | Urethral secretion/swab | 0.012 | 0.016 | 0.016 | -0.42 | Yes | 33 | S | S |
| NG007 | m | 24 | Urethral secretion/swab | 0.012 | 0.016 | 0.032 | -1.42 | Yes | 33 | S | S |
| NG008 | m | 43 | Urethral secretion/swab | 0.012 | 0.016 | 0.016 | -0.42 | Yes | 35 | S | S |
| NG009 | f | 54 | Vaginal secretion/swab | 0.012 | 0.016 | 0.016 | -0.42 | Yes | 36 | S | S |
| NG010 | m | 22 | Urethral secretion/swab | 0.012 | 0.016 | 0.032 | -1.42 | Yes | 35 | S | S |
| NG011 | m | 55 | Urethral secretion/swab | 0.016 | 0.016 | 0.016 | 0.00 | Yes | 36 | S | S |
| NG012 | m | 41 | Urethral secretion/swab | 0.094 | 0.125 | 0.125 | -0.41 | Yes | 30 | S | S |
| NG013 | m | 41 | Urethral secretion/swab | 0.064 | 0.064 | 0.125 | -0.97 | Yes | 30 | S | S |
| NG014 | m | 20 | Urethral secretion/swab | 0.012 | 0.016 | 0.016 | -0.42 | Yes | 39 | S | S |
| NG015 | m | 21 | Urethral secretion/swab | 0.012 | 0.016 | 0.016 | -0.42 | Yes | 38 | S | S |
| NG016 | m | 52 | Urethral secretion/swab | 0.012 | 0.016 | 0.016 | -0.42 | Yes | 35 | S | S |
| NG017 | m | 46 | Urethral secretion/swab | 0.012 | 0.016 | 0.016 | -0.42 | Yes | 32 | S | S |
| NG018 | m | 26 | Urine | 0.064 | 0.064 | 0.064 | 0.00 | Yes | 30 | S | S |
| NG019 | m | 39 | Urethral secretion/swab | 0.064 | 0.064 | 0.125 | -0.97 | Yes | 32 | S | S |
| NG020 | m | 37 | Urethral secretion/swab | 0.012 | 0.016 | 0.016 | -0.42 | Yes | 30 | S | S |
| NG021 | m | 19 | Urethral secretion/swab | 0.25 | 0.25 | 0.5 | -1.00 | Yes | 28 | I | R |
| NG022 | m | 37 | Urethral secretion/swab | 0.25 | 0.25 | 0.25 | 0.00 | Yes | 26 | I | I |
| NG023 | m | 33 | Urethral secretion/swab | 0.094 | 0.125 | 0.125 | -0.41 | Yes | 27 | S | S |
| NG024 | m | 35 | Urethral secretion/swab | 0.25 | 0.25 | 0.25 | 0.00 | Yes | 38 | I | I |
| NG025 | m | 30 | Urethral secretion/swab | 0.125 | 0.125 | 0.125 | 0.00 | Yes | 31 | S | S |
| NG026 | m | 22 | Urethral secretion/swab | 0.008 | 0.008 | 0.008 | 0.00 | Yes | 37 | S | S |
| NG027 | m | 20 | Urethral secretion/swab | 0.125 | 0.125 | 0.125 | 0.00 | Yes | 32 | S | S |
| NG028 | m | 41 | Urethral secretion/swab | 0.094 | 0.125 | 0.125 | -0.41 | Yes | 29 | S | S |
| NG029 | m | 32 | Urine | 0.006 | 0.008 | 0.004 | 0.58 | Yes | 34 | S | S |
| NG030 | m | 39 | Urethral secretion/swab | 0.008 | 0.008 | 0.008 | 0.00 | Yes | 36 | S | S |
| NG031 | m | 33 | Urethral secretion/swab | 0.006 | 0.008 | 0.004 | 0.58 | Yes | 40 | S | S |
| NG032 | m | 23 | Urine | 0.016 | 0.016 | 0.016 | 0.00 | Yes | 31 | S | S |
| NG033 | m | 32 | Urethral secretion/swab | 0.125 | 0.125 | 0.125 | 0.00 | Yes | 30 | S | S |
| NG034 | m | 18 | Urethral secretion/swab | 0.012 | 0.016 | 0.016 | -0.42 | Yes | 38 | S | S |
| NG035 | m | 35 | Urethral secretion/swab | 0.012 | 0.016 | 0.016 | -0.42 | Yes | 35 | S | S |
| NG036 | m | 26 | Urethral secretion/swab | 0.125 | 0.125 | 0.125 | 0.00 | Yes | 32 | S | S |
| NG037 | f | 36 | Vaginal secretion/swab | 0.125 | 0.125 | 0.125 | 0.00 | Yes | 33 | S | S |
| NG038 | m | 28 | Urethral secretion/swab | 0.125 | 0.125 | 0.125 | 0.00 | Yes | 33 | S | S |
| NG039 | m | 22 | Urethral secretion/swab | 0.125 | 0.125 | 0.125 | 0.00 | Yes | 34 | S | S |
| NG040 | m | 22 | Urethral secretion/swab | 0.125 | 0.125 | 0.125 | 0.00 | Yes | 31 | S | S |
| NG041 | m | 21 | Urine | 0.064 | 0.064 | 0.125 | -0.97 | Yes | 33 | S | S |
| NG042 | m | 21 | Urethral secretion/swab | 0.064 | 0.064 | 0.064 | 0.00 | Yes | 33 | S | S |
| NG043 | m | 65 | Urethral secretion/swab | 0.012 | 0.016 | 0.016 | -0.42 | Yes | 36 | S | S |
| NG044 | m | 49 | Urethral secretion/swab | 0.75 | 1 | 1 | -0.42 | Yes | 21 | R | R |
| NG045 | m | 27 | Urethral secretion/swab | 0.012 | 0.016 | 0.016 | -0.42 | Yes | 36 | S | S |
| NG046 | m | 23 | Urethral secretion/swab | 0.5 | 0.5 | 0.5 | 0.00 | Yes | 27 | R | R |
| NG047 | m | 57 | Urethral secretion/swab | 0.25 | 0.25 | 0.25 | 0.00 | Yes | 28 | I | I |
| NG048 | m | 26 | Urethral secretion/swab | 0.25 | 0.25 | 0.5 | -1.00 | Yes | 25 | I | R |
| NG049 | m | 43 | Urethral secretion/swab | 0.012 | 0.016 | 0.032 | -1.42 | Yes | 36 | S | S |
| NG050 | m | 34 | Urethral secretion/swab | 0.125 | 0.125 | 0.125 | 0.00 | Yes | 30 | S | S |
| NG051 | m | 50 | Urethral secretion/swab | 0.25 | 0.25 | 0.5 | -1.00 | Yes | 28 | I | R |
| NG052 | m | 71 | Urethral secretion/swab | 0.5 | 0.5 | 0.5 | 0.00 | Yes | 20 | R | R |
| NG053 | m | 21 | Urethral secretion/swab | 0.5 | 0.5 | 0.5 | 0.00 | Yes | 22 | R | R |
| NG054 | f | 19 | Vaginal secretion/swab | 0.125 | 0.125 | 0.125 | 0.00 | Yes | 28 | S | S |
| NG055 | m | 44 | Urethral secretion/swab | 0.054 | 0.064 | 0.064 | -0.25 | Yes | 37 | S | S |
| NG056 | m | 16 | Urethral secretion/swab | 0.012 | 0.016 | 0.016 | -0.42 | Yes | 38 | S | S |
| NG057 | m | 44 | Urethral secretion/swab | 0.024 | 0.032 | 0.064 | -1.42 | Yes | 42 | S | S |
| NG058 | m | 20 | Urethral secretion/swab | 0.094 | 0.125 | 0.125 | -0.41 | Yes | 35 | S | S |
| NG059 | m | 37 | Urethral secretion/swab | 0.125 | 0.125 | 0.125 | 0.00 | Yes | 27 | S | S |
| NG060 | f | 32 | Vaginal secretion/swab | 0.048 | 0.064 | 0.064 | -0.42 | Yes | 36 | S | S |
| NG061 | m | 19 | Urethral secretion/swab | 0.38 | 0.5 | 0.5 | -0.40 | Yes | 36 | R | R |
| NG062 | m | 71 | Urethral secretion/swab | 0.012 | 0.016 | 0.032 | -1.42 | Yes | 43 | S | S |
| NG063 | m | 20 | Urethral secretion/swab | 0.012 | 0.016 | 0.032 | -1.42 | Yes | 38 | S | S |
| NG064 | f | 26 | Vaginal secretion/swab | 0.25 | 0.25 | 0.25 | 0.00 | Yes | 30 | I | I |
| NG065 | m | 42 | Urethral secretion/swab | 0.008 | 0.008 | 0.008 | 0.00 | Yes | 31 | S | S |
| NG066 | m | 26 | Urethral secretion/swab | 0.25 | 0.25 | 0.25 | 0.00 | Yes | 30 | I | I |
| NG067 | f | 53 | Vaginal secretion/swab | 0.006 | 0.008 | 0.008 | -0.42 | Yes | 36 | S | S |
| NG068 | m | 35 | Urethral secretion/swab | 0.25 | 0.25 | 0.25 | 0.00 | Yes | 29 | I | I |
| NG069 | m | 19 | Urethral secretion/swab | 0.012 | 0.016 | 0.016 | -0.42 | Yes | 37 | S | S |
| NG070 | m | 53 | Urine | 0.5 | 0.5 | 1 | -1.00 | Yes | 28 | R | R |
| NG071 | m | 27 | Urine | 0.38 | 0.5 | 0.5 | -0.40 | Yes | 39 | R | R |
| NG072 | m | 31 | Urethral secretion/swab | 0.006 | 0.008 | 0.004 | 0.58 | Yes | 41 | S | S |
| NG073 | m | 20 | Urethral secretion/swab | 0.008 | 0.008 | 0.008 | 0.00 | Yes | 38 | S | S |
| NG074 | m | 25 | Urethral secretion/swab | 0.006 | 0.008 | 0.008 | -0.42 | Yes | 35 | S | S |
| NG075 | m | 22 | Urethral secretion/swab | 0.012 | 0.016 | 0.032 | -1.42 | Yes | 35 | S | S |
| NG076 | f | 60 | Urine | 0.012 | 0.016 | 0.016 | -0.42 | Yes | 35 | S | S |
| NG077 | m | 22 | Urethral secretion/swab | 0.012 | 0.016 | 0.016 | -0.42 | Yes | 31 | S | S |
| NG078 | m | 27 | Urethral secretion/swab | 0.006 | 0.008 | 0.008 | -0.42 | Yes | 35 | S | S |
| NG079 | m | 30 | Urethral secretion/swab | 0.064 | 0.064 | 0.064 | 0.00 | Yes | 32 | S | S |
| NG080 | m | 35 | Urethral secretion/swab | 0.012 | 0.016 | 0.016 | -0.42 | Yes | 41 | S | S |
| NG081 | m | 20 | Urethral secretion/swab | 0.012 | 0.016 | 0.016 | -0.42 | Yes | 36 | S | S |
| NG082 | f | 21 | Vaginal secretion/swab | 0.012 | 0.016 | 0.016 | -0.42 | Yes | 37 | S | S |
| NG083 | m | 43 | Urine | 0.012 | 0.016 | 0.016 | -0.42 | Yes | 37 | S | S |
| NG084 | m | 41 | Urethral secretion/swab | 0.012 | 0.016 | 0.032 | -1.42 | Yes | 30 | S | S |
| NG085 | m | 33 | Urethral secretion/swab | 0.008 | 0.008 | 0.008 | 0.00 | Yes | 35 | S | S |
| NG086 | m | 31 | Urethral secretion/swab | 0.5 | 0.5 | 0.5 | 0.00 | Yes | 32 | R | R |
| NG087 | m | 59 | Urethral secretion/swab | 0.012 | 0.016 | 0.016 | -0.42 | Yes | 38 | S | S |
| NG088 | f | 28 | Vaginal secretion/swab | 0.064 | 0.064 | 0.064 | 0.00 | Yes | 30 | S | S |
| NG089 | m | 30 | Urethral secretion/swab | 0.25 | 0.25 | 0.5 | -1.00 | Yes | 29 | I | R |
| NG090 | m | 22 | Urine | 0.064 | 0.064 | 0.064 | 0.00 | Yes | 23 | S | S |
| NG091 | m | 38 | Urethral secretion/swab | 0.5 | 0.5 | 0.5 | 0.00 | Yes | 24 | R | R |
| NG092 | m | 32 | Urethral secretion/swab | 0.25 | 0.25 | 0.5 | -1.00 | Yes | 29 | I | R |
| NG093 | f | 62 | Vaginal secretion/swab | 0.5 | 0.5 | 0.5 | 0.00 | Yes | 24 | R | R |
| NG094 | m | 19 | Vaginal secretion/swab | 0.38 | 0.5 | 0.5 | -0.40 | Yes | 36 | R | R |
| NG095 | m | 22 | Urine | 0.064 | 0.064 | 0.064 | 0.00 | Yes | 35 | S | S |
| NG096 | f | 63 | Pus | 0.094 | 0.125 | 0.125 | -0.41 | Yes | 33 | S | S |
| NG097 | m | 53 | Urethral secretion/swab | 0.125 | 0.125 | 0.125 | 0.00 | Yes | 33 | S | S |
| NG098 | m | 27 | Urethral secretion/swab | 0.125 | 0.125 | 0.125 | 0.00 | Yes | 35 | S | S |
| NG099 | m | 40 | Urethral secretion/swab | 0.094 | 0.125 | 0.125 | -0.41 | Yes | 33 | S | S |
| NG100 | m | 20 | Urethral secretion/swab | 0.006 | 0.008 | 0.008 | -0.42 | Yes | 43 | S | S |
| NG101 | m | 35 | Urethral secretion/swab | 0.25 | 0.25 | 0.5 | -1.00 | Yes | 28 | I | R |
| NG102 | m | 30 | Urethral secretion/swab | 0.25 | 0.25 | 0.25 | 0.00 | Yes | 29 | I | I |
| NG103 | m | 23 | Urethral secretion/swab | 0.25 | 0.25 | 0.25 | 0.00 | Yes | 29 | I | I |
| NG104 | m | 34 | Urethral secretion/swab | 0.19 | 0.25 | 0.25 | -0.40 | Yes | 32 | I | I |
| NG105 | m | 20 | Urethral secretion/swab | 0.125 | 0.125 | 0.125 | 0.00 | Yes | 33 | S | S |
| NG106 | m | 22 | Urethral secretion/swab | 0.38 | 0.5 | 0.5 | -0.40 | Yes | 35 | R | R |
| NG107 | m | 52 | Urethral secretion/swab | 0.094 | 0.125 | 0.125 | -0.41 | Yes | 27 | S | S |
| NG108 | m | 29 | Urethral secretion/swab | 0.125 | 0.125 | 0.125 | 0.00 | Yes | 28 | S | S |
| NG109 | m | 24 | Urethral secretion/swab | 0.032 | 0.032 | 0.064 | -1.00 | Yes | 31 | S | S |
| NG110 | m | 60 | Urethral secretion/swab | 0.25 | 0.25 | 0.25 | 0.00 | Yes | 28 | I | I |
| NG111 | m | 29 | Urethral secretion/swab | 0.064 | 0.064 | 0.125 | -0.97 | Yes | 28 | S | S |

Table S1 note. The table is deidentified. Gradient strip MIC testing refers to the Autobio Diagnostics ceftriaxone gradient strip product; agar dilution was the reference method. The rounded MIC column is used for essential agreement, categorical agreement, error rates, and S/I/R categorization. The raw log2 difference uses the original continuous gradient reading. The revised CLSI display convention maps the experimental 0.125 mg/L dilution to ≤0.12 mg/L; S = susceptible (≤0.125 mg/L), I = intermediate (0.25 mg/L), and R = resistant (≥0.5 mg/L).

Table S2. Raw continuous gradient strip readings that differed from agar dilution by more than one twofold dilution before upward rounding.

| **Study ID** | **Gradient raw MIC** | **Gradient rounded MIC** | **Agar dilution MIC** | **Raw log2 difference** | **Rounded log2 difference** | **Gradient category** | **Agar category** |
| --- | --- | --- | --- | --- | --- | --- | --- |
| NG003 | 0.012 | 0.016 | 0.032 | -1.42 | -1.00 | S | S |
| NG007 | 0.012 | 0.016 | 0.032 | -1.42 | -1.00 | S | S |
| NG010 | 0.012 | 0.016 | 0.032 | -1.42 | -1.00 | S | S |
| NG049 | 0.012 | 0.016 | 0.032 | -1.42 | -1.00 | S | S |
| NG057 | 0.024 | 0.032 | 0.064 | -1.42 | -1.00 | S | S |
| NG062 | 0.012 | 0.016 | 0.032 | -1.42 | -1.00 | S | S |
| NG063 | 0.012 | 0.016 | 0.032 | -1.42 | -1.00 | S | S |
| NG075 | 0.012 | 0.016 | 0.032 | -1.42 | -1.00 | S | S |
| NG084 | 0.012 | 0.016 | 0.032 | -1.42 | -1.00 | S | S |

Table S2 note. These nine records were non-essential by the raw continuous reading, but all were within ±1 dilution after upward rounding, remained susceptible by both methods, and did not undergo categorical reclassification.

Table S3. Isolates with complete CLSI 2026 S/I/R categorical discrepancies after standardized upward rounding.

| **Study ID** | **Gradient rounded MIC** | **Agar dilution MIC** | **Gradient category** | **Agar category** | **Error type** |
| --- | --- | --- | --- | --- | --- |
| NG021 | 0.25 | 0.5 | I | R | Minor error (gradient I / agar R) |
| NG048 | 0.25 | 0.5 | I | R | Minor error (gradient I / agar R) |
| NG051 | 0.25 | 0.5 | I | R | Minor error (gradient I / agar R) |
| NG089 | 0.25 | 0.5 | I | R | Minor error (gradient I / agar R) |
| NG092 | 0.25 | 0.5 | I | R | Minor error (gradient I / agar R) |
| NG101 | 0.25 | 0.5 | I | R | Minor error (gradient I / agar R) |

Table S3 note. Six minor errors were observed; no major or very major errors occurred. The categories use S = susceptible, I = intermediate, and R = resistant under CLSI M100, 36th edition (2026).
